# The experiential perspectives of parents caring for a loved one with a restrictive eating disorder in the UK

**DOI:** 10.1192/bjo.2021.1019

**Published:** 2021-10-12

**Authors:** Hannah Cribben, Pamela Macdonald, Janet Treasure, Erica Cini, Dasha Nicholls, Rachel Batchelor, Carol Kan

**Affiliations:** Institute of Psychiatry, Psychology & Neuroscience, King's College London, UK; Institute of Psychiatry, Psychology & Neuroscience, King's College London, UK; Institute of Psychiatry, Psychology & Neuroscience, King's College London, UK; East London Eating Disorder Service for Children and Young People, East London NHS Foundation Trust, UK; and Nutrition Science Group, Division of Medicine, University College London, UK; Division of Psychiatry, Imperial College London, UK; Royal Holloway, Department of Psychology, University of London, UK; Institute of Psychiatry, Psychology & Neuroscience, King's College London, UK

**Keywords:** Carers, anorexia nervosa, bulimia nervosa, eating disorders not otherwise specified, patients

## Abstract

**Background:**

Parents of a loved one with an eating disorder report high levels of unmet needs. Research is needed to understand whether clinical guidance designed to improve the experience of parents has been effective.

**Aims:**

To establish parents’ experiential perspectives of eating disorder care in the UK, compared with guidance published by Beat, a UK eating disorders charity, and Academy for Eating Disorders, the leading international eating disorders professional association.

**Method:**

A total of six focus groups (one online and five face-to-face) were held throughout the UK. A total of 32 parents attended. All participants were parents of a loved one with a diagnosis of anorexia nervosa or atypical anorexia nervosa (mean age 22 years; mean duration of illness 4.4 years). Focus groups were transcribed, and the text was analysed with an inductive approach, to identify emerging themes.

**Results:**

Four key themes were identified: (a) impact of eating disorder on one's life, (b) current service provisions, (c) navigating the transition process and (d) suggestions for improvement.

**Conclusions:**

Current experiences of parents in the UK do not align with the guidelines published by Beat and Academy of Eating Disorders. Parents identified a number of changes that healthcare providers could make, including improved information and support for parents, enhanced training of professionals, consistent care across all UK service providers, policy changes and greater involvement of families in their loved one's care. Findings from this project informed the design of a national web-survey on loved ones’ experience of care in eating disorders.

It is estimated that over 1.6 million people in the UK are affected by eating disorders. As the age at onset of an eating disorder is usually before the individual has left home,^1^ the role of caregiver often falls to the parents. Research has demonstrated that the involvement of close others in all stages of care can have a positive impact on treatment outcomes.^2^ However, the parental role can be challenging; a systematic review identified perceived increased burden and low efficacy for carers, with both associated with clinical levels of depression and anxiety.^3^ There is some evidence that this can negatively affect treatment outcomes in people with eating disorders.^4^ Furthermore, carers and close others often report a lack of adequate support or information during their loved one's illness,^5–8^ particularly if the person with the eating disorder is legally an adult. In response to these unmet needs, guidance has been issued by both the Academy for Eating Disorders (AED),^9^ the leading international association for eating disorder professionals, and Beat, a UK eating disorder charity,^10^ highlighting recommendations and guidance to meet the needs of families and carers affected by eating disorders (see Table 1). More research is needed to understand the extent to which experiences of care for parents throughout the UK meet the standards outlined in the guidance, and what changes could be implemented to improve their experience of care and reduce levels of concern and distress, thereby potentially improving outcomes for patients with eating disorders. We aimed to develop a greater understanding of parents’ experiential perspective of eating disorder treatment in the UK, compared with these published guidelines, by conducting face-to-face/online focus groups. Additional focus groups were organised for partners and siblings; these findings will be explored in a separate paper. The findings from this project will inform the design of a national web-survey on loved ones’ experience of care in eating disorders.

**Table 1:** Published guidelines for meeting the needs of families and carers affected by eating disorders

Academy for Eating Disorders
1. Access to quality care: All patients have the right to immediate care for medical and/or psychiatric instability, followed by timely and non-discriminatory access to appropriate specialty care.
2. Respect: All patients, caregivers and family members have the right to be treated with respect throughout the assessment, planning and treatment process. Patients and carers should never be judged or stigmatised based on symptoms, behaviours or past treatment history.
3. Informed consent: When making healthcare decisions, patients and caregivers have the right to full disclosure by healthcare professionals about treatment best-practices, risks, costs, expected service outcomes, other treatment options, and the training and expertise of their clinicians.
4. Participation: Families and other designated carers have a right to participate in treatment as advocates for the best interests of their loved ones. Caregiving responsibilities and degrees of participation will necessarily vary depending on the age, mental state and diagnosis of the patient, as well as the caregiver's skills, availability, personal health, resources and other circumstances.
5. Communication: All patients and carers have the right to establish regular and ongoing communications through clearly defined channels. Caregivers and family members have the right to communicate their observations and concerns to professionals and to receive information when the patient's medical stability and/or psychiatric safety is threatened or at risk.
6. Privacy: All patients and carers have a right to expect their health professionals to understand, communicate and respect the applicable privacy or age-of-consent regulations that govern the communication of health and treatment information, as well as the circumstances and conditions that may override privacy concerns or transfer authority regarding treatment decisions.
7. Support: All caregivers have a right to receive information, resources and support services to help them understand and carry out the expectations and responsibilities of their roles as partners in treatment.
Beat
1. Have a policy that ensures optimum involvement of and support for all carers as soon as a loved one starts treatment.
2. Train all service staff in the application of the policy and these standards with particular focus on the importance of carers as a resource for recovery.
3. Provide all carers with useful and comprehensive information about eating disorders when their loved one receives a diagnosis.
4. Offer all carers and siblings an assessment of their own needs when a loved one receives an eating disorder diagnosis, continue to monitor their well-being throughout the sufferer's treatment and, where necessary, refer carers to specialist services.
5. Offer all carers options for peer-to-peer support.
6. Offer all carers opportunities to learn the necessary skills to provide optimum support for their loved ones.
7. Inform and engage carers when a loved one faces a transition between services and ensure that effective communication between both services and carers takes place.
8. Provide a mechanism by which carers’ input and feedback is sought and acted upon.

## Method

### Participants and sampling

Parents of individuals with an eating disorder were recruited across the UK via advertisements by local and national eating disorder charities. Parents were excluded if their loved one had not received eating disorder treatment in the UK within the past 10 years. Thirty-two parents (twenty-four women, eight men) participated overall. The majority (*n* = 19; 59%) were aged between 51 and 60 years and of White ethnicity (96.9%). Two couples, each caring for one child with an eating disorder, attended the focus groups.

The ages of the parents’ loved one with an eating disorder ranged from 13 to 45 years, with a mean of 22.00 years (s.d. 7.33); ten (33.3%) were aged <18 years, and twenty (66.6%) were aged ≥18 years. Most of the individuals with an eating disorder were female (*n* = 28; 93.3%), with a diagnosis of anorexia nervosa (*n* = 28; 93.3%) or atypical anorexia nervosa (*n* = 2; 6.7%). The duration of illness ranged from 6 months to 15 years, with an average duration of 4.40 years (s.d. 3.59); 16 (53.3%) had an illness duration of <3 years, and 14 (46.7%) had a more enduring illness (i.e. duration of >3 years). Twenty-six (86.7%) of these individuals had experience of out-patient care, seventeen (56.7%) had accessed in-patient care and three (10%) had received day-care treatment. Eight (26.7%) had only ever accessed child and adolescent mental health services (CAMHS) treatment settings, eight (26.7%) only had experience of care in adult services and 14 (46.7%) had accessed both CAMHS and adult services.

### Procedure

This study was performed in accordance with the Declaration of Helsinki and approved by King's College London (approval number HR-19/20-14803). All participants provided written informed consent to participate. All focus groups were held early in 2020; five were face-to-face focus groups held in London, Derby, Wrexham and Belfast, to ensure cross-UK representation. In light of the COVID-19 outbreak, the sixth focus group was held virtually, using Microsoft Teams for Mac (Version 16.35); this group was exclusively open to parents who had experienced care in Scotland.

A topic guide for the focus groups was collaboratively developed in advance during two discussion groups of professionals, carers and patients. At the start of each focus group, participants completed demographic questionnaires. The focus groups were led by one of two researchers (C.K. or H.C.), with the lead researcher recording the discussion. During the focus groups, the participants were informed of the overall aims of the research, and the recommendations published by both the AED and Beat were outlined to aid the discussion. The topic guide used in all focus groups is outlined in Appendix 1. Recordings were transcribed, and any identifiable information was removed before analysis.

### Analysis

Data were analysed by using an inductive approach to thematic analysis at the semantic level, meaning that themes are identified within the explicit meanings of the data, and analysed and reported without trying to fit into a pre-existing coding frame, or the researcher's preconceptions.^11^ Two researchers (P.M. and H.C.) worked independently on the identification of themes within the transcripts, following the six phases outlined by Braun and Clarke.^11^ When devising the final thematic framework, researchers held discussions on eight separate occasions, with themes being consolidated and labels edited to more accurately reflect the content of the nodes after each discussion. All analysis was carried out with NVivo for Mac (Version 12, QSR International, Doncaster, Australia; see https://www.qsrinternational.com/nvivo-qualitative-data-analysis-software/support-services/nvivo-downloads).

## Results

**Fig. 1:**
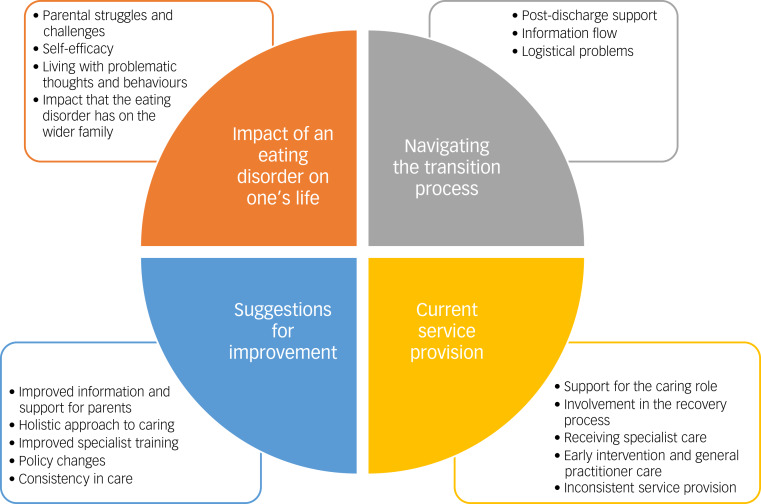
Higher- and lower-order themes generated from focus groups, using thematic analysis.

Four higher-order themes emerged from the six data-sets, outlined alongside the lower-order themes in Fig. 1.

### Impact of an eating disorder on one's life

Parents described their perspectives of how their child's eating disorder has affected life for themselves and their family. Accounts of parental struggles and challenges included confusion, frustration, guilt, stress, loneliness, feeling overwhelmed, hopelessness, lack of control and fear for the future. Parents reflected upon perceived mistakes made in their caring responses and the stigma faced throughout the illness. Parents described noticing deteriorations in their own physical/mental health since the onset of the illness, and highlighted financial/logistical barriers to accessing support for this.

‘And you can feel terribly isolated from your kind of friends …’ Focus group 3, participant 6 (female).

‘I realise a lot of what I was doing wasn't actually helping him …’ Focus group 2, participant 3 (female).

‘I'm going on to antidepressants at the end of the month because it's just too stressful …’ Focus group 4, participant 1 (female).

Parent and patient self-efficacy was highlighted in all focus groups. Examples of parent self-efficacy included efforts and determination to access appropriate care and/or information, as well as efforts to improve the quality of care provided. Further comments related to parent's attempts to protect their own well-being, such as adopting a positive mindset. Parents described the perceived benefits of their loved one also exhibiting self-efficacy throughout the treatment journey.

‘I got access to information by finding out people whose children are going through it and really directly contacting the tertiary services, you know, the regional services and asking for help.’ Focus group 2, participant 1 (female).

‘But apart from the one appointment that I took her to, she's driven herself to all the appointments, I've had no contact whatsoever and she has done very well …’ Focus group 5, participant 1 (female).

Parents described living with problematic thoughts and behaviours, including their perceptions of their loved one's rigid eating patterns, body shape concerns and age-inappropriate thoughts and behaviours. The worries encountered by parents when they perceive their loved one to be resisting and/or denying treatment were also described.

‘… she's 27 but she's behaving like a child, she calls me mummy …’ Focus group 1, participant 2 (female).

‘… this time, she's got that body dysmorphia which she didn't have the last time, so she doesn't see the bones are sticking out of her.’ Focus group 4, participant 3 (male).

Parents expressed concern for the impact that the eating disorder has on the wider family including siblings, partners or the wider family system.

‘We used to have loads of friends come over all the time, and then my sons … stopped inviting friends round because they didn't know whether plates would be thrown, or she would yell.’ Focus group 5, participant 3 (female).

### Current service provisions

The following subthemes reflect parent's accounts on any aspect of service provision. Parents reflected on the availability and accessibility of support for the caring role, including information, skills training, peer support, family therapy and meal support. Methods of support provision were described, including through service providers, charities/external organisations, schools, online platforms/social media or other carers. Further comments described the quality and usefulness of the support provided.

‘I found the family therapy quite good because it's a really good environment for us to chat in, with having someone else there to sort of act as a mediator.’ Focus group 3, participant 4 (female).

‘The parent support group that we go to, that was my lifeline, and they have more skill, more knowledge, more idea of what you're going through as a family because they've been there.’ Focus group 5, participant 5 (female).

Parents reflected upon their level of involvement in the recovery process. There are accounts of parents feeling excluded from the recovery process because of confidentiality policies, intentional or unintentional actions of the clinical team, or the patient themselves not wanting their carers involved. Further comments related to an exclusion from service provision more generally, in terms of not seeking or valuing carer's feedback. Some positive experiences of feeling included in the recovery process are also covered in this node.

‘… if we ask a question we're definitely not being blanked. Those questions are being absorbed and being answered’ Focus group 1, participant 1 (male).

‘We didn't have any feedback from appointments, we had no contact with the service at all and felt like we were absolutely left hanging.’ Focus group 6, participant 2 (female).

Carers reflected upon their loved one's experiences relating to receiving specialist care for the eating disorder. There are descriptions of problems receiving high-quality health and social care for various reasons, including strict admission criteria and discharge policies, inadequate training of staff, stigma, bed/staff/financial shortages, extensive waiting lists, administrative errors and/or unsatisfactory adjustments made for patients with comorbidities. Carers reflected on the consequences of delayed or inadequate access to services. A smaller number of comments reflected positively on the quality of care delivered by specialist teams.

‘Our daughter asked to be admitted and she was told no, today, she doesn't meet the criteria.’ Focus group 4, participant 1 (female).

‘… she was put on the urgent list because her BMI [body mass index] was so low but that was 3 months waiting list’ Focus group 5, participant 5 (female).

‘I know where I am, once you're referred to the eating disorder service they will see you within a week and they are really good.’ Focus group 2, participant 3 (female).

Parents described experiences of early intervention and general practitioner (GP) care, including the quality of care received from GPs during early consultations. Parents emphasised the importance of early intervention and reflected upon the adequacy of information and support given to patients and their families up to and including the diagnostic assessment. Although most comments described negative experiences, some parents reflected positively on the early stages of accessing care.

‘… she presented herself to the doctors and said ‘I think I've got an eating disorder’. They weighed her and said ‘well you're not, you're too heavy’ so there was no referral made, there was no suggestion or anything, it was almost dismissed.’ Focus group 1, participant 3 (female).

‘the GP was really good and referred her really fast.’ Focus group 5, participant 3 (female).

There are many references to inconsistent service provision, including disparities between services either within the same area (e.g. CAMHS versus adults; day care versus community care) or across different geographical areas. This node also incorporated inconsistences in care within the same service, such as conflicting advice from staff. Parents described disparities between treatment provisions for physical illnesses such as cancer, compared with mental illnesses.

‘They're meant to be governed by the NICE [National Institute for Health and Care Excellence] guidelines, aren't they? So, they all should be providing the same thing, but they're not’ Focus group 4, participant 2 (male).

### Navigating the transition process

There were several subthemes that represented issues concerning the transition process from in-patient to home or CAMHS to adult services. Parents described views on post-discharge support offered to either patient, their families or both, including follow-up and relapse prevention plans, community support and crisis planning. The majority of these views were negative, although there were a small number of positive experiences.

‘… she sees a community nurse once a week, she comes to the house, and she was told that there was no care plan, there hadn't been one for the last 6 months. So the treatment she's receiving is totally inadequate or even, can't even think of the word, non-existent.’ Focus group 3, participant 1 (female).

‘… the hospital supported her to get some work experience, she loves dogs and so she's been going to puppy training, that's the first time she's engaged in anything …’ Focus group 1, participant 3 (female).

Furthermore, the transition process can be affected by information flow, with problems reported in communication and sharing of information regarding the transition process causing considerable confusion. This node also featured a small number of positive comments relating to helpful communication between teams during the transition process.

‘The counsellor she was seeing, or the therapist, did say they hadn't forwarded her on any information so she was dealing with it in the dark and I think that encouraged our daughter to think “what's the point, every time I go I have to start all over again I won't do it”.’ Focus group 1, participant 2 (female).

‘… I was in that handover meeting, and things were discussed’ Focus group 5, participant 2 (female).

Finally, parents also describe how logistical problems involving geographical, funding or time constraint can affect the transition process.

‘… we had the experience when we moved from one trust to another, we had to apply for special funding and then that was us to write to the commissioners, I mean we're lucky we could do it’ Focus group 3, participant 4 (female).

### Suggestions for improvement

This theme incorporates carers’ ideas and suggestions as to what improvements could be made to improve experiences for themselves and their loved one. Parents suggested a perceived need for improved information and support for parents, including increased education and information regarding eating disorders and service provision, alongside a need for reassurance that the parents are not to blame. Suggestions for improved support came in various formats, from a community care coordinator to greater support from the team itself, enhanced therapeutic provisions, skills training, guidance with meal times, support for the carer's own emotional well-being and tailored support depending on the carer's relationship to their loved one.

‘… every single person should have a care coordinator who is responsible for pulling everything together.’ Focus group 1, participant 4 (female).

‘… even if they just gave you a pack each on diagnosis, leaflets, information, they don't even have to tell you anything, just a ready-made pack to go.’ Focus group 3, participant 1 (female).

Throughout all six focus groups, there were references to the desire for a more holistic approach to caring. Quotes indicate the desire for greater inclusion of close others in their loved one's treatment as well as being made to feel more valued in the process. Parents wanted to encourage the healthcare system to tailor treatments to the family structure, such as considering the needs of siblings or separated/divorced parents. There were further suggestions to include the patient's wider network, including schools.

‘… whether it's in CAMHS or adult, in-patient or out-patient, they need to be listening to the parents and understand that the parents know their child best.’ Focus group 3, participant 2 (female).

‘CAMHS I think should involve schools as well for their input.’ Focus group 5, participant 5 (female).

Parents also expressed the necessity of improved specialist training and resources for GPs to refer to appropriate specialist teams. Parents expressed an additional need for improved and more widespread specialist training for those who are providing treatment for the eating disorder.

‘… well-trained staff would be the priority’ Focus group 3, participant 5 (female).

Parents outlined their desire for policy changes, including changes to the legal framework, improved funding (especially for adult services), reduced use of bank staff, changes to discharge policies, addressing the present ‘confidentiality’ issue, implementing a system that provides carers with more input after patients are ≥18 years of age and the eradication of body mass index (BMI) cut-off values required to access services.

‘… I don't need to know everything that's been said but perhaps if she's been given release of hospital to our care, we need to know a little about what's happened.’ Focus group 2, participant 4 (female).

‘I would stop BMI [body mass index], because I think it's outdated …’ Focus group 5, participant 3 (female).

Finally, parents described the importance of consistency in care and the need to ensure a consistent and standardised approach to care for all patients, across the UK, and over time. Comments related to the benefits of having consistency in the healthcare professional(s) and support systems that a patient and their family access throughout their treatment.

‘It should be a standardisation of services and information for carers that is given to every carer as a basic minimum from every service in the country …’ Focus group 2, participant 1 (female).

## Discussion

The main aim of this study was to better our understanding of parents’ experiential perspective of eating disorder treatment in the UK, compared with published guidelines, to inform the design of a national survey for carers. Some parents did reflect upon positive experiences of care in the UK, matching the standards outlined in published guidelines. For example, some parents felt confident that they could ask questions about their loved one's treatment, aligning with Beat's eighth recommendation (see Table 1). The AED's first recommendation is that all patients are given access to quality care, and some parents in our sample felt that this was achieved in terms of referral and/or waiting list durations (see section ‘Current service provisions’). Additionally, there were examples of appropriate transition support for carers and their loved ones, including offering work experience to support transition into the community and inviting carers to a hand-over meeting when transferring between teams (see section ‘Navigating the transition process’). These are practical examples of how service providers can meet Beat's seventh recommendation regarding transition support. Finally, both the AED and Beat emphasise the importance of providing support for the caring role, and our findings highlight some of the many ways that this has been accessed, including through family therapy and peer support (see section ‘Current service provisions’).

However, crucially, our findings revealed a number of shortcomings in current service provisions for parents of those with an eating disorder at all stages of treatment. This builds upon our understanding from previous research,^5–8,12^ as we now know that a lack of adequate support or information for parents is a common experience for parents across the UK, in a variety of treatment settings and at all treatment stages. Therefore, although some parents described positive experiences of care in the UK, it remains clear that standards are yet to reach the levels outlined in published guidelines.

### Clinical implications

To improve parent's experiences and meet the standards outlined in the Beat and AED published guidelines, parents in our focus groups suggested a number of practical steps that service providers can take. Some of these suggestions were based on experiences of what had worked well, whereas others were based on negative experiences of care. The consensus was that, overall, improvements were required to meet the standard of care outlined by Beat and the AED. Parents would greatly benefit from improved information and support, as well as healthcare professionals adopting a more inclusive approach to parents. Both the Beat and AED guidelines recommend involving carers at all stages of treatment; this is of particular importance as we know that this can have positive effects on their loved ones’ treatment outcomes.^13^ Our findings suggest that not all parents currently feel involved in their loved ones’ treatment (see section ‘Current service provisions’), and this is something that parents would value (see section ‘Suggestions for improvement’). However, parents expressed a desire to address the current ‘confidentiality’ issue with regards to patients aged ≥18 years. Although the right to confidentiality applies equally to adults and children, the difference in CAMHS settings is that the parent also has a right to care for their child (and hence requires the necessary knowledge to perform this care); this may explain why confidentiality appeared to be a more pertinent issue within adult settings. The AED specifically states that healthcare providers must ‘respect the applicable privacy or age-of-consent regulations that govern the communication of health and treatment information’.^9^ Therefore, it is important that healthcare providers, especially in adult services, explore methods of ensuring that parents are involved in the recovery process without breaching confidentiality guidelines.

Previous research has demonstrated how such an approach can be beneficial for carers; the New Maudsley collaborative care intervention provides carers with theoretical and practical knowledge for coping with eating disorders.^14^ This approach has been successful both in the form of a workshop^15^ and self-help guides,^16,17^ highlighting how a change in approach could target parents’ currently unmet needs. Alongside this, a manualised psychoeducational group for early-onset eating disorders in children and adolescents could address parent's desire for information on eating disorders alongside access to peer support, and can feasibly be delivered in a UK healthcare setting.^18^ Further practical steps that healthcare providers could realistically take include ensuring that all services have easily accessible resources for parents/carers, having a named person that the parent can contact and involving parents early in their child's treatment journey.

Work is also being done to address the need for improved specialist training. In England, a national whole-team training (WTT) for children and young people's eating disorders services (CYP EDS) was delivered to all CYP EDS in 2017, as part of the implementation of the Access and Waiting Times Standard for Children and Young People with an Eating Disorder Commissioning Guide.^19^ A similar WTT for adult eating disorder services in England was commissioned for delivery in 2021.^20^ In addition, first-episode rapid early intervention for eating disorders (FREED^18^) is a recently developed service model and care package that has been implemented within the UK healthcare system. Pilot studies indicate that adopting this model leads to reduced waiting times^21^ and improved clinical outcomes.^22^ Over the next 5 years, it is hoped that FREED will be scaled up to be available to all young people who need it, and become more established in Wales, Scotland and Northern Ireland.^23^

Although these examples illustrate how the healthcare system can make practical changes to improve parents’ current experiences, our national survey will seek to explore these ideas in greater detail, so that suggestions for improvement can be strengthened by their specificity.

### Limitations

Participants in our sample were included if their child had accessed care for an eating disorder any time in the past 10 years, and therefore may have experienced care before the AED and Beat guidelines were published – in 2017 and 2019, respectively – making it difficult to ascertain the level of influence that these guidelines have had. However, our analysis did not reveal any perceived improvements in the quality of service provisions over time (i.e. since the guidelines were published), and the large majority of our participants were not aware that the guidelines existed. Although we measured illness duration, we did not specifically measure time since diagnosis; this would have been helpful to assess whether there were variations in participant's experiences dependent upon whether they had received care in recent years compared with approximately 10 years ago.

Furthermore, almost half of our sample had accessed eating disorder care in both CAMHS and adult services. Because of the anonymisation of transcripts before analyses, it was not possible to conduct a direct comparison between CAMHS and adult services, making it difficult to establish whether our findings are equally relevant in both settings. Parents are routinely involved in treatment for young people <16 years of age, and parents of adolescent anorexia nervosa patients report high levels of satisfaction with specialist services,^24^ which may influence responses in some areas. Furthermore, CAMHS eating disorder services have undergone significant transformation since the introduction of increased funding in 2016,^25^ and therefore there may be further disparities in experiences of care dependent on whether an individual received care before or after 2016. Future research should seek to investigate the impact of service transformation and associated training on adherence to the Beat and AED published guidelines on carers’ involvement and experiences.

Our study was interrupted by the COVID-19 outbreak and subsequent lockdown, with the final focus group being moved online. Our analysis did not reveal any significant differences between the experiences of parents in the Scotland focus group (post-COVID-19) and previous focus groups (pre-COVID-19), suggesting that the experience of care for those in the Scotland focus group was not significantly affected by the pandemic. However, this indifference may be attributable to the small sample size in Scotland (*n* = 4) compared with all other focus groups combined (*n* = 28), or because the topic guide was not altered in light of COVID-19 to directly assess the effects of the lockdown. A qualitative study specifically exploring the effects of lockdown for patients with anorexia nervosa and their carers indicated that COVID-19 has presented unique challenges and some increased practical demands for carers^26^; it would have been helpful to explore this explicitly in our focus groups.

Although we did reach data saturation with our sample size, our sample lacked diversity, with most parents being White, middle-aged women caring for their adult child with an enduring restrictive eating disorder. Furthermore, 56.7% of our sample had accessed in-patient care, suggesting an illness severity on the severe end of the spectrum. Therefore, these findings may not be generalisable to all cases of eating disorders. Nonetheless, we do know that these findings are relevant in all areas of the UK (at least for some individuals), as we recruited participants in England, Wales, Scotland and Northern Ireland. We aim to increase the diversity of our sample by widening access to this research via our national online survey. In addition, by involving carers in all aspects of our research from informing the topic guide for focus groups to analysing the results, we hope that our study design and findings are relevant to the large number of parents affected by eating disorders throughout the UK.

As with all qualitative research, there is possible research bias. However, this was reduced by using an independent researcher (P.M.), who had not attended the focus groups or been involved in the study design, to code the transcripts alongside a second researcher (H.C.), who had attended the focus groups. Finally, although the current paper did not address the experiences of other close others/carers, such as siblings and partners, the research team have explored this in a separate study, which will be published later this year.

## Data Availability

The data that support the findings of this study are available from the corresponding author, H.C., upon reasonable request. The data are not publicly available due to containing information that could compromise the privacy of research participants.

## Appendix 1: Focus Group Topic Guide

### Introduction and overview

- Welcome and introductions to research team
- Overview of the aims of the study: ‘Our overall aim is to develop a national survey to improve understanding of the experience of close others of patients with eating disorders. From doing so, we hope to identify areas of improvement. Today, we will be using your experiences to help us to develop this survey. We will be using 2 sets of recommendations outlined by BEAT, the UK charity of eating disorders, and the Academy for Eating Disorders. We would like to know whether your lived experiences align with these guidelines. This will inform the development of our national survey.’
- Instructions regarding the focus group: ‘There is no right or wrong answers – all your responses are valid. Please give everyone a balanced use of airtime. Please help to protect others’ privacy by not discussing details outside of today's focus group. Please don't use identifiable information in the discussions (e.g., your loved one's name). We are recording the discussion – once you have said something, you cannot erase it from the recording so please only share what you are comfortable with. Please try to stay on topic – we only have one hour!’

### Materials

All participants are provided a pack of materials, which includes a consent form, information sheet, participant demographics form, copy of the Beat recommendations and copy of the Academy for Eating Disorder recommendations.

### Discussion points (approximately 10 min per discussion point)

- Which recommendations are you aware of or familiar with?
- Focusing on the recommendations from Beat, please choose the top 3 most important recommendations
- Looking at the top 3 recommendations:
  - What would you consider good support for carers of people with eating disorders?
  - How do they match your personal experience?
- What other good practise for carers have you experienced?
- What do you want out of the national survey?

## References

[1] Micali N, Hagberg KW, Petersen I, Treasure JL. The incidence of eating disorders in the UK in 2000–2009: findings from the General Practice Research Database. BMJ Open 2013; 3(5): e002646. 10.1136/bmjopen-2013-002646 PMC3657659 23793681

[2] Le Grange D, Lock J, Loeb K, Nicholls D. Academy for eating disorders position paper: the role of the family in eating disorders. Int J Eat Disord 2010; 43(1): 1–5. 19728372 10.1002/eat.20751

[3] Anastasiadou D, Medina-Pradas C, Sepulveda AR, Treasure J. A systematic review of family caregiving in eating disorders. Eat Behav 2014; 15(3): 464–77. 25064301 10.1016/j.eatbeh.2014.06.001

[4] Zabala MJ, Macdonald P, Treasure J. Appraisal of caregiving burden, expressed emotion and psychological distress in families of people with eating disorders: a systematic review. Eur Eat Disord Rev 2009; 17(5): 338–49. 19367608 10.1002/erv.925

[5] Winn S, Perkins S, Murray J, Murphy R, Schmidt U. A qualitative study of the experience of caring for a person with bulimia nervosa. Part 2: carers’ needs and experiences of services and other support. Int J Eat Disord 2004; 36(3): 269–79. 15478136 10.1002/eat.20068

[6] Perkins S, Winn S, Murray J, Murphy R, Schmidt U. A qualitative study of the experience of caring for a person with bulimia nervosa. Part 1: the emotional impact of caring. Int J Eat Disord 2004; 36(3): 256–68. 15478131 10.1002/eat.20067

[7] Graap H, Bleich S, Herbst F, Trostmann Y, Wancata J, de Zwaan M. The needs of carers of patients with anorexia and bulimia nervosa. Eur Eat Disord Rev 2008; 16(1): 21–9. 18074327 10.1002/erv.804

[8] Mitrofan O, Petkova H, Janssens A, Kelly J, Edwards E, Nicholls D, Care experiences of young people with eating disorders and their parents: qualitative study. BJPsych Open 2019; 5(1): e6. 30762506 10.1192/bjo.2018.78 PMC6343117

[9] Academy for Eating Disorders. *World Eating Disorder Healthcare Rights. An AED Global Blueprint for Promoting Excellence in Care through Patient-Carer-Professional Partnerships.* Academy for Eating Disorders, 2017 (https://higherlogicdownload.s3.amazonaws.com/AEDWEB/27a3b69a-8aae-45b2-a04c-2a078d02145d/UploadedImages/Advocate/World_ED_Rights_Flyer_04_03_2017.pdf).

[10] Beat. *Best Practice in the Engagement and Empowerment of Families and Carers Affected by Eating Disorders.* Beat, 2019 (https://allcatsrgrey.org.uk/wp/download/public_health/mental_health/family-empowerment-guidance.pdf).

[11] Braun V, Clarke V. Qualitative research in psychology using thematic analysis in psychology using thematic analysis in psychology. Qual Res Psychol 2006; 3(2): 77–101.

[12] Schmit SE, Bell NJ. Close relationships and disordered eating: partner perspectives. J Health Psychol 2017; 22(4): 434–45. 26349618 10.1177/1359105315603478

[13] Le Grange D, Fitzsimmons-Craft EE, Crosby RD, Hay P, Lacey H, Bamford B, Predictors and moderators of outcome for severe and enduring anorexia nervosa. Behav Res Ther 2014; 56: 91–8. 24727364 10.1016/j.brat.2014.03.006

[14] Treasure J, Rhind C, Macdonald P, Todd G. Collaborative care: the new Maudsley model. Eat Disord 2015; 23(4): 366–76. 26010051 10.1080/10640266.2015.1044351

[15] Pépin G, King R. Collaborative care skills training workshops: helping carers cope with eating disorders from the UK to Australia. Soc Psychiatry Psychiatr Epidemiol 2013; 48(5): 805–12. 22961291 10.1007/s00127-012-0578-6

[16] Hibbs R, Magill N, Goddard E, Rhind C, Raenker S, Macdonald P, Clinical effectiveness of a skills training intervention for caregivers in improving patient and caregiver health following in-patient treatment for severe anorexia nervosa: pragmatic randomised controlled trial. BJPsych Open 2015; 1(1): 56–66. 27703724 10.1192/bjpo.bp.115.000273 PMC4998946

[17] Goddard E, Raenker S, Macdonald P, Todd G, Beecham J, Naumann U, Carers’ assessment, skills and information sharing: theoretical framework and trial protocol for a randomised controlled trial evaluating the efficacy of a complex intervention for carers of inpatients with anorexia nervosa. Eur Eat Disord Rev 2013; 21(1): 60–71. 22961838 10.1002/erv.2193

[18] Rosello R, Gledhill J, Yi I, Watkins B, Harvey L, Hosking A, Early intervention in child and adolescent eating disorders: the role of a parenting group. Eur Eat Disord Rev 2021; 29(3): 519–26. 33084198 10.1002/erv.2798

[19] NHS England. *Access and Waiting Time Standard for Children and Young People with an Eating Disorder: Commissioning Guide*. NHS England, 2015 (https://www.basw.co.uk/system/files/resources/basw_25346-5_0.pdf).

[20] NHS England. *Adult Eating Disorder Whole Team Training Curriculum – December 2019.* NHS England, 2019 (https://www.hee.nhs.uk/sites/default/files/documents/Adult%20eating%20disorder%20whole%20team%20training.pdf).

[21] Brown A, McClelland J, Boysen E, Mountford V, Glennon D, Schmidt U. The FREED project (first episode and rapid early intervention in eating disorders): service model, feasibility and acceptability. Early Interv Psychiatry 2018; 12(2): 250–7. 27619198 10.1111/eip.12382

[22] McClelland J, Hodsoll J, Brown A, Lang K, Boysen E, Flynn M, A pilot evaluation of a novel first episode and rapid early intervention service for eating disorders (FREED). Eur Eat Disord Rev 2018; 26(2): 129–40. 29460477 10.1002/erv.2579

[23] Allen KL, Mountford V, Brown A, Richards K, Grant N, Austin A, First episode rapid early intervention for eating disorders (FREED): from research to routine clinical practice. Early Interv Psychiatry 2020; 14(5): 625–30. 32064736 10.1111/eip.12941

[24] Roots P, Rowlands L, Gowers SG. User satisfaction with services in a randomised controlled trial of adolescent anorexia nervosa. Eur Eat Disord Rev 2009; 17(5): 331–7. 19548250 10.1002/erv.944

[25] NHS England. *The Five Year Forward View for Mental Health*. NHS England, 2016 (https://www.england.nhs.uk/wp-content/uploads/2016/02/Mental-Health-Taskforce-FYFV-final.pdf).

[26] Clark Bryan D, Macdonald P, Ambwani S, Cardi V, Rowlands K, Willmott D, Exploring the ways in which COVID -19 and lockdown has affected the lives of adult patients with anorexia nervosa and their carers. Eur Eat Disord Rev 2020; 28(6): 826–35. 32643844 10.1002/erv.2762 PMC7362064

